# Efficacy and safety of heat-sensitive moxibustion in the treatment of neurogenic bladder after spinal cord injury: A protocol for systematic review and meta-analysis

**DOI:** 10.1097/MD.0000000000026424

**Published:** 2021-06-18

**Authors:** Qianqian Lin, Yafeng Ren, Kewei Chen, Huijie Duan, Meng Chen, Chengmei Liu

**Affiliations:** a Henan University of Chinese Medicine; b The First Affiliated Hospital of Henan University of Chinese Medicine, Zhengzhou, Henan, China.

**Keywords:** heat-sensitive moxibustion, neurogenic bladder, protocol, spinal cord injury, systematic review

## Abstract

**Background:**

Spinal cord injury (SCI) is one of the most disabling and destructive neurological diseases. Neurogenic bladder dysfunction (NBD) is one of the serious complications after SCI, 80% of patients after SCI will have neurogenic bladder symptoms. NBD after SCI may lead to urinary retention, urinary incontinence, and urinary tract infection. In severe cases, it can lead to renal failure or even death. NBD after SCI not only seriously affects the patient's quality of life but also physical and mental health. NBD after SCI is a social and medical problem. In recent years, more and more clinical studies prove that heat-sensitive can improve the clinical symptoms of NBD after SCI. Therefore, this article conducts a systematic evaluation and meta-analysis on the efficacy and safety of heat-sensitive moxibustion in treating NBD after SCI.

**Methods:**

Search 8 electronic databases including PubMed, Embase, Web of Science, The Cochrane Library, Clinical Trials, China National Knowledge Infrastructure, China Science and Technology Journal Database, Wanfang Database, and China Biomedical Literature Database. We will search above electronic databases from the inception to May 2021, without any language restriction. Clinical randomized controlled trials containing heat-sensitive moxibustion for NBD after SCI and eligible interventions(s) and outcome(s) were included, with no limitation of language and publication status. Two researchers will independently conduct literature search, screening, information extraction, quality assessment, and data analysis. Review Manager 5.3 software will be used for statistical analysis.

**Results:**

The findings will be submitted to a peer-reviewed publication.

**Conclusion:**

This systematic review and meta-analysis will provide a standard clinical decision-making guideline for heat-sensitive moxibustion treatment of NBD after SCI.

**INPLASY registration number:**

INPLASY202150071.

## 1 Introduction

Spinal cord injury (SCI) is one of the most disabling and destructive neurological diseases.^[1]^ The clinical outcomes of SCI depend on the severity and location of the lesion. SCI includes partial or complete loss of sensory and/or motor function below the level of injury.^[2]^ According to the World Health Organization, about 250,000 to 500,000 people around the world sustain an SCI each year.^[3]^ The peak age of SCI is about 38 years old, and there are more men than women. Falling from a height is the main cause of SCI, followed by car accident injuries.^[4]^ Neurogenic bladder dysfunction (NBD) is one of the serious complications after SCI.^[5]^ SCI patients have a 80% chance of developing NBD.^[6]^ Based on the locations of the spinal cord lesions, the NBD can be classified as upper motor neuron dysfunctions (injuries above S1) and lower motor neuron dysfunctions (injuries at S1–S4).^[6]^ Different segments and degrees of SCI can lead to different types NBD, which will lead to different clinical manifestations.^[7]^ NBD after SCI may lead to urinary retention, urinary incontinence, and urinary tract infection. In severe cases, it can lead to renal failure which is the first cause of death in patients with SCI in China.^[8,9]^

A study has shown that 39.0% of NBD patients require a urology visit, 33.3% were hospitalized, and 14.4% were in a nursing home.^[10]^ The disease brings physical pain to patients and causes heavy economic and psychological burden.^[11]^ According to reports, the annual cost of rehospitalization of patients with SCI due to urinary tract infection reached 16 million in the United States.^[12]^ It is a social and medical problem. Early intervention, correct treatment, and regular follow-up can avoid complications and improve the quality of life of patients with NBD due to SCI to the greatest extent.^[13]^

Nowadays, NBD cannot be completely cured. The primary goal of its treatment is to protect kidney function and enable patients to survive for a long time; the secondary goal is to improve the quality of life of patients with NBD due to SCI.^[14]^ Clean intermittent catheterization is considered as the gold standard to manage urinary symptoms related to NBD.^[13]^ However, the evidence of the best intermittent urinary catheterization method (including cleaning and disinfection methods, catheter materials, catheterization frequency, etc) is still insufficient.^[15]^ Invasive surgery not only brings many risks and complications but also has limitations. Western medicine treatment has not only low expectations and many side effects but also high consumption, which will increase the burden of family and society.

With the widespread of Traditional Chinese Medicine in the world, acupuncture and moxibustion is more and more popular.^[16]^ In 1979, the World Health Organization drafted out a provisional list of 47 diseases that could be treated by means of acupuncture therapy, including NBD following SCI.^[17,18]^ Although the mechanism of acupuncture and moxibustion treatment NBD after SCI is not yet clear, but it is certain that acupuncture can activate nerve regeneration in damaged nerves, stimulate and/or regulate nerve conduction to regulate the physiological function of the bladder, and promote the recovery of bladder function.^[19,20]^ Heat-sensitive moxibustion is a new type of acupuncture and moxibustion which emphasizes the selection of acupoint and uses moxa heat to stimulate heat-sensitive acupoints. It has the characteristics of simplicity, convenience, cheapness, and effectiveness in clinical ^[21]^. In recent years, more and more clinical studies prove that heat-sensitive moxibustion can improve the clinical symptoms of NBD after SCI.^[22–28]^ However, the efficacy and safety of heat-sensitive moxibustion in treating NBD after SCI have not been systematically evaluated. Therefore, this article will systematically evaluate the efficacy and safety of heat-sensitive moxibustion in the treatment of NBD after SCI.

## 2 Method and analysis

### 2.1 Study registration

This protocol has been registered with the International Platform of Registered Systematic Review and Meta-Analysis Protocols on 18 May 2021. The registration number is INPLASY 202150071. We will conduct this protocol following the guidelines of Cochrane Handbook for Systematic Reviews of Interventions and the Preferred Reporting Items to conduct this Systematic Reviews and Meta-analysis Protocol (PRISMA-P) statement.^[29]^

### 2.2 Eligibility criteria

#### 2.2.1 Types of study

Clinical randomized controlled trials (RCTs) containing heat-sensitive moxibustion for NBD after SCI were included, with no limitation of language and publication status. Case reports, nonrandomized clinical studies, quasi-RCTs, reviews, and animal studies will be excluded.

#### 2.2.2 Types of participants

All patients with NBD after SCI (as diagnosed by a clinician, or using any recognized diagnostic criteria) will be included without any restrictions, such as race, age, and gender.

#### 2.2.3 Types of interventions

##### 2.2.3.1 Experimental interventions

Heat-sensitive moxibustion therapy or mixed therapies based on heat-sensitive moxibustion will also be included.

##### 2.2.3.2 Control interventions

The control group was treated with conventional treatment and rehabilitation or combined with other acupuncture but except heat-sensitive moxibustion.

#### 2.2.4 Types of outcomes

##### 2.2.4.1 Primary outcome

Clinical efficacy including total effective rate and a voiding diary (the mean number of urination or incontinence episodes per 24 hours, the number of participants with incontinence or retention, and the number of participants requiring catheterisation) was reported by participants.

##### 2.2.4.2 Secondary outcomes

1. Changes in urodynamic tests before and after treatment, for example, maximum urinary flow rate (Qmax), postvoiding residual urine volume, maximal detrusor, pressure, urethral closure pressure, effective bladder capacity.
2. Changes in a QoL questionnaire before and after treatment.
3. Safety outcome measures such as the incidence of all reported adverse events.

### 2.3 Search strategy

Studies will be searched in 8 electronic databases: PubMed, Embase, Web of Science, The Cochrane Library, Clinical Trials, China National Knowledge Infrastructure, Chinese Science and Technology Periodical Database, Wanfang Database, and Chinese Biomedical Literature Database. We will search above electronic databases from the inception to May 2021, without any language restriction. We will also trace the references of relevant studies to ensure that any potential eligible RCTs will not be missed. The PubMed search strategy is recorded in Table 1. We will provide similar search strategies in other Chinese and English databases.

**Table 1:** The search strategy for PubMed.

Number	Search terms
#	Search strategy
1	“Spinal Cord Injuries”[Mesh]
2	Spinal cord injury[Title/Abstract] OR spinal cord traum[Title/Abstract] OR spinal cord contusion[Title/Abstract] OR spinal cord lesion[Title/Abstract] OR paraplegia[Title/Abstract]
3	#1 OR #2
4	“Urinary Bladder, Neurogenic”[Mesh]
5	neurogenic bladder[Title/Abstract] OR bladder disorder[Title/Abstract] OR uninhibited bladder[Title/Abstract] OR bladder dysfunction[Title/Abstract] OR emiction disorder[Title/Abstract] OR aconuresis[Title/Abstract] OR incontinence[Title/Abstract] OR retention[Title/Abstract] OR uroschesis[Title/Abstract]
6	#4 OR #5
7	Heat-sensitive moxibustion[Title/Abstract] OR Thermal moxibustion[Title/Abstract] OR Thermosensitive hanging moxibustion[Title/Abstract] OR heat-sensitive acupoint[Title/Abstract] OR Heat sensitizing acupoint moxibustion[Title/Abstract] OR new moxibustion[Title/Abstract] OR heat sensitization[Title/Abstract]
8	#3 AND #6 AND #7

### 2.4 Data collection and analysis

#### 2.4.1 Selection of studies

**Figure 1:**
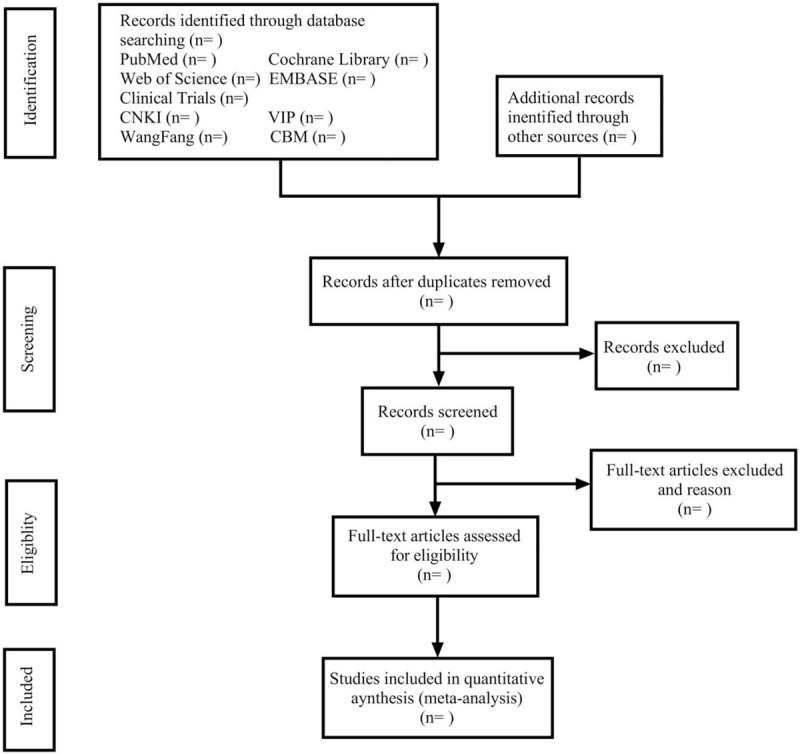
Flow chart of the study selection.

Two reviewers will independently search the literature according to the predetermined search strategy; the results of search will be imported into EndNote X9 software. After the elimination of duplicates, the 2 reviewers will identify the literature that may meet the eligibility criteria by browsing the titles and abstracts of the preliminarily screened literature. Then, by reading the full text of all eligible studies to decide whether a trial will be included. At the same time, the 2 reviewers will carry out crosschecking and extract relevant information and data. If there is any disagreement, the third author will join in the discussion and consultation. A flowchart (Fig. 1) will be used to depict the selection process of the study.

#### 2.4.2 Data extraction and management

The data extraction will be conducted by 2 reviewers, respectively. The following data will be extracted and recorded on the upfront excel sheet: the first author, publication year, country, sample size, treatment measures, study design and methods, assessment time, follow-up time, and outcome index. Any divergence will be resolved by discussion; if the results of the discussion cannot be unified, a third reviewer will be invited.

#### 2.4.3 Assessment of risk of bias

The quality evaluation of the included study will be conducted by 2 reviewers using Cochrane collaboration's tool, including sequence generation, allocation concealment, blinding of participants and assessors, blinding of outcome assessment, incomplete outcome data, selective reporting, and other bias, which can evaluate the risk of bias of the final included studies. Each item rated as low-risk, unclear, and high-risk, and consulted a third reviewer when necessary.

#### 2.4.4 Data analysis

We will use Review Manager 5.3 software to perform data analysis. The heterogeneity will be evaluated by Cochran's *Q* test and Higgins *I*^2^ statistic between the included studies. If there is no statistical heterogeneity (*P* > .1, *I*^2^ < 50%), fixed-effect model will be used for meta-analysis. Otherwise, random-effect model will be used after the source of heterogeneity was excluded. All outcomes will be analyzed using 95% confidence intervals (95% CI).

#### 2.4.5 Subgroup analysis

If there is a significant heterogeneity in the studies, subgroup analysis will be carried out according to different factors based on the type of NBD, treatment cycle, each moxibustion treatment time, and the type of intervention in the control group.

#### 2.4.6 Sensitivity analysis

The purpose of sensitivity analysis is to determine the sources and confounding factors of heterogeneity. If the trial data is sufficient, low or high quality studies will be excluded one by one for sensitivity analysis.

#### 2.4.7 Assessment of reporting biases

If there are more than 10 articles, a funnel plot would be will be performed, which used to demonstrate publication bias by its symmetry.^[30]^

#### 2.4.8 Evaluation of the evidence quality

Two reviewers will respectively assess the evidence quality of each study by applying the Grading of Recommendations Assessment, Development and Evaluation system.^[31]^ The quality of evidence will be divided into high, moderate, low, and very low.

#### 2.4.9 Ethics and dissemination

Because this study does not involve private patient information, no ethical review is required. The results of this system review and meta-analysis will be published in a peer-reviewed journal.

## 3 Discussion

NBD after SCI can be classified as “ischuria” in Traditional Chinese Medicine. Ischuria is closely related to the kidney, spleen, lung, triple-burner, and bladder. Moxibustion helps to warm meridian and dredge collaterals, harmonize qi and blood, nourish liver and kidney, and promote the function of bladder qi transformation. Modern research suggests that the curative effect of moxibustion is the result of a comprehensive effect of multiple factors. It not only has a stimulating effect, but also has physical characteristics such as heat and light. The light radiation produced by the burning of mugwort leaves acts on acupoint, causing skin cells to produce inflammation; which achieve the role of regulating the body.^[32]^ As a new type of moxibustion therapy, heat-sensitive moxibustion can treat NBD after SCI by heat sensitized acupoints in the bladder area of the abdomen, so as to achieve the purpose of treatment^[22]^. This systematic review and meta-analysis will summarize the most recent RCTs, to determine the efficacy and safety of heat-sensitive moxibustion for NBD due to SCI. We hope that this study will provide evidence for the clinical treatment of patients with NBD due to SCI.

## Author contributions

**Conceptualization:** Qianqian Lin, Yafeng Ren, Kewei Chen.

**Data curation:** Huijie Duan, Meng Chen.

**Formal analysis:** Qianqian Lin, Kewei Chen.

**Funding acquisition:** Yafeng Ren, Chengmei Liu.

**Methodology:** Yafeng Ren, Qianqian Lin, Meng Chen.

**Project administration:** Chengmei Liu.

**Resources:** Kewei Chen, Huijie Duan.

**Software:** Qianqian Lin, Meng Chen.

**Supervision:** Chengmei Liu, Yafeng Ren.

**Writing – original draft:** Qianqian Lin, Yafeng Ren.

**Writing – review & editing:** Qianqian Lin, Huijie Duan.
